# ASAS-NANP symposium: mathematical modeling in animal nutrition: contributions of mathematical modeling to life cycle assessment to support environmental sustainability of animal production

**DOI:** 10.1093/jas/skaf442

**Published:** 2025-12-18

**Authors:** Alice Cadéro, Luis Orlindo Tedeschi, Florence Garcia-Launay

**Affiliations:** PEGASE, INRAE, Institut-Agro, 16 Le Clos, Saint-Gilles, 35590, France; Department of Animal Science, Texas A&M University, College Station, TX 77843-2471; PEGASE, INRAE, Institut-Agro, 16 Le Clos, Saint-Gilles, 35590, France

**Keywords:** environmental impact, individual-based models, livestock production, mitigation strategies, process-based models

## Abstract

Life cycle assessment (LCA) provides a standardized framework for evaluating the environmental impacts of animal production systems through its four key steps: 1) goal and scope definition, 2) inventory analysis, 3) impact assessment, and 4) interpretation. However, traditional approaches using surveys and experimental data face limitations in capturing complex interactions among biological processes and management practices. This paper reviews how mathematical modeling can enhance LCA methodology for animal production systems, overcoming these constraints and supporting more robust environmental impact assessments. Mathematical models contribute significantly to LCA methodology at multiple scales and stages. At the inventory analysis stage, models predict feed intake, growth, production, and excretion of nutrients in response to animal characteristics and management practices. These range from nutritional metabolic models of average animals to sophisticated individual-based models that account for variability among animals in a herd. A systematic workflow could be followed for developing stochastic, individual-based models that generate comprehensive life cycle inventories through a bottom-up approach. Process-based models also improve emission estimates from animals and manure, progressing from simple Tier 1 default emission factors to complex Tier 3 mechanistic approaches that capture interactions between management practices and environmental factors. However, significant challenges remain in modeling manure emissions due to complex data requirements and microbial dynamics. Beyond inventory development, mathematical modeling enhances LCA’s utility for decision support through optimization models that identify mitigation strategies balancing environmental and economic objectives. Individual-based models enable environmental phenotyping for genetic selection by quantifying how individual animal traits affect system-level impacts. These approaches represent promising developments for sustainable livestock production. Mathematical modeling transforms LCA from a descriptive tool to a predictive framework capable of evaluating numerous scenarios across different production contexts. Further development should focus on integrating performance and emission models, implementing optimization approaches for mitigation strategy identification, and expanding applications to regional and national scales to support evidence-based policies.

## Teaser

Mathematical modeling revolutionizes life cycle assessment of livestock systems by capturing complex biological interactions that are invisible to traditional methods. This integration enables precise evaluation of environmental impacts from feeding strategies, genetics, and management practices across scales from individual animals to entire production sectors. The synergy between process-based models, optimization techniques, and emerging precision livestock technologies creates powerful new frameworks for designing sustainable animal production systems.

## Introduction

It is widely accepted that animal production has various detrimental effects on the environment. On a global scale, livestock production systems are responsible for 13% to 14.5% of total GHG anthropogenic emissions (Gerber et al., 2013; Herrero et al., 2016). They also need large land areas for grazing and production of diet ingredients, including cereals and forages (Mottet et al., 2017). They are a driver of land-use change and, consequently, biodiversity loss, as illustrated in various regions worldwide. In many areas of the world, specialized production systems (livestock and crop) and territories result in strong imports of synthetic fertilizers and diet ingredients. As a result, N and P are available in excess, recycling through organic fertilization remains low, and emissions of pollutants occur at different steps of manure management (Billen et al., 2015, 2018). Livestock production systems contribute to acidification mainly through ammonia emissions and to the eutrophication of freshwater and coastal zones through nitrates and phosphates leaching after field application of manure (McAuliffe et al., 2016). They contribute to depriving nonrenewable or limited resources like inorganic P (Geissler et al., 2019), fossil energy, and natural resources like fresh water (Mekonnen and Hoekstra, 2012). Consequently, animal production is a key driver of humanity’s offense of several planetary boundaries (Bowles et al., 2019). Drastic changes in these systems and farming practices are required to address and reduce the livestock sector’s environmental impacts. Various levers and combinations adapted to the pedoclimatic conditions should be investigated and assessed thoroughly.

Life cycle assessment (LCA) provides an internationally recognized standardized method to evaluate the environmental impacts of any agricultural production process, and substantial efforts have been made to harmonize its application to animal production systems (FAO, 2016a, 2018). After defining the goal and scope of the study, it inventories all the resources consumed and all the pollutants emitted associated with the life cycle of a production process, and it converts them into environmental impacts through characterization methods. Many LCA of animal production processes have been performed, and several reviews abound in the literature (Baldini et al., 2017; Andretta et al., 2021; Gislason et al., 2023). Cradle-to-farm gate LCA are the most common studies available in the literature since on-farm and upstream processes have the highest contributions to animal products’ environmental impacts. They provide an overview of the environmental impacts, their hotspots, and a range of values for each animal species. They also offer insights into the effectiveness of mitigation options applied to farming systems.

To date, many LCA studies in animal production rely on surveys and per or experimental data, combined with emission factors, either fixed or context and technology-specific equations, to calculate the emissions in closed buildings, grazing paddocks, open feedlot pens, and manure storage. Therefore, the results of LCA studies largely depend on the location of experimental conditions and suffer the usual lack of genericity of experimental studies. These approaches do not make it possible to account for the effects of the interactions between factors, and in these cases, the results seldom allow support for decisions and changes in system management. In particular, the accuracy of the emissions inventory is a special challenge given the complexity and uncertainty inherent to biological processes and their management occurring in livestock production systems. There are also numerous controversies regarding methodological issues in LCA. For instance, some studies criticize LCA for its capacity to encompass the effects of mitigation strategies to support decisions (Faverdin et al., 2022) or for the difficulty in addressing the multifunctionality of processes and allocating impacts among co-products (Wilfart et al., 2021). The functional unit is also somewhat questionable, even if the kilogram of live weight at the farm gate is the most usual.

There is a long history of mathematical modeling in animal production systems to support decisions for animal management, feeding and breeding strategies, diet formulation, and optimization, among many others. In his recent review, Tedeschi (2023) summarized why mathematical modeling can be effective in understanding and predicting the environmental impacts of animal production systems. In particular, process-based models make it possible to understand the link between biotechnical processes and their subsequent effects on the environment (consumption of resources, emissions of pollutants). Since they aim to represent the relevant components of the system under study and their interactions, they improve the understanding of the overall responses of those systems (Jones et al., 2017; Tedeschi, 2023). Therefore, they can help identify management options for improving environmental sustainability. In addition, they also make it possible to forecast environmental impacts in future conditions (e.g., climate change).

The limitations of the LCA methodology could be overcome using process-based modeling. We contend that associating mathematical modeling and LCA is necessary to support the development of environmentally sustainable livestock farming systems. Many studies have already associated mathematical modeling of animal production systems with LCA in the last decade. Therefore, this paper proposes an overview of the potential added values of associating mathematical modeling and LCA with animal production to overcome the limitations of LCA. It will cover three main topics: 1) the main steps of LCA in animal production and the methodological challenges associated, 2) the specific contribution of mathematical modeling to inventory analysis, and 3) the use of mathematical modeling and LCA to support mitigation of impacts. This review will include process-based models that cover the usual perimeters of cradle-to-farm gate LCA in animal production or part of the perimeter and predict animal performance and per or nutrient excretion, emissions at housing, storage, and pasture. It will also focus on models that can test mitigation options, considering various factors like feeding strategies, genetics, and manure management. It will not consider emissions models at field application or during manure treatment since FAO (2016a, 2018) recommends cutting off system boundaries after manure storage to treat manure as a residue beneficial to crop. It will also exclude models predicting the effects of various disease challenges (e.g., pathogens, mastitis) and their consequences on environmental impacts.

## Overview of the Main Steps of LCA in Animal Production and Methodological Challenges

**Figure 1.**
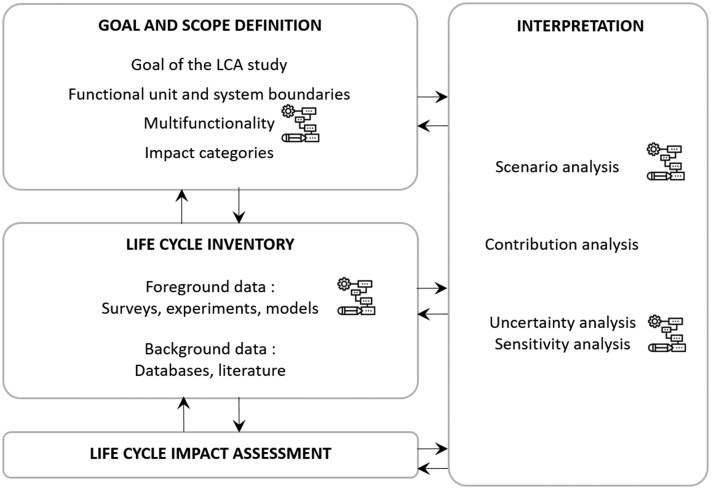
Schematic of ISO 14044 stages of life cycle assessment with reference to the topics developed in this review (adapted from FAO, 2018). The pictograms indicate the areas of contribution of mathematical modeling in animal production.

Life cycle assessment is an internationally recognized standardized method, described with an ISO standard (ISO, 2006), and its application to animal production systems is described in various guidelines (FAO, 2016a, 2016b, 2016c, 2018). Life cycle assessment is a four-step method (Figure 1). It is applied into four main steps, namely goal and scope, inventory analysis, life cycle impact assessment, and interpretation. For each of these steps, we examine the advantages of process-based modeling within LCA, particularly in relation to the methodological limitations of traditional approaches (Table 1); further details are provided below.

**Table 1.** Comparative analysis of limitations (italic) and advantages of traditional life cycle assessment (LCA) and process-based modelling associated to LCA

Traditional LCA approach	Process-based modelling associated to LCA
**Goal and scope definition**	
*Not possible to apply a biophysical allocation of the impacts between co-products*	Ability to apply a biophysical allocation using processes simulation
**Life cycle inventory**	
*Dependency on local conditions of the measured data and lack of genericity*	Ability to apply the approach for various local conditions in order to gain genericity
*Potential biases and uncertainties in measurements*	*Potential biases uncertainties in model parameters and gaps in processes represented*
*Use of global references for data unavailable through measurements*	Access to data unavailable through measurements (e.g., methane conversion factor)
Usually available data sufficient for this approach	*Complex data requirements and parameterization issues for mechanistic models*
**Life cycle impact assessment**	
**Not applicable**	
**Interpretation**	
*Not possible to assess the effects of interactions between factors*	Virtual experiments to assess the effects of interactions between factors
*Uncertainty and sensitivity analyses available only for parameters of the LCI without consideration of their interactions*	Uncertainty and sensitivity analyses while considering internal processes and their interactions
*Indirect link between farm management, animal characteristics, and resulting environmental impacts*	Explicit link between farm management, animal characteristics, and resulting environmental impacts
*Limited ability to support decision making*	Ability to support investigation of mitigation strategies and decision making through optimization

### First step—goal and scope

Various goals can be targeted in animal production systems: eco-design, product labeling, and comparison of conventional and alternative production systems. Most LCA studies in animal production are cradle-to-farm gate LCA. According to the goal, defining system boundaries and internal flows and components is necessary. Most LCA of animal production systems include crop production and processing to obtain diet ingredients; the output of non-plant feed ingredients like feed-grade amino acids, enzymes, and minerals; and the inputs of these processes (electricity, seeds, fuel, agriculture machinery). They also include feed fabrication, all the transport steps (transport of feed ingredients and feeds), and on-farm processes.

At this stage, it is also necessary to define the primary function of the production system to define the functional unit that will be used to express the environmental impacts. Various functional units have been used in animal production systems LCA depending on the study’s objectives, the data available, and the type of production system: kg of live weight at farm gate, kg of live weight gain during fattening, ha of land used, kg of milk. The issue of defining an adequate function unit is not fully solved in the literature. Most cradle-to-farm gate studies use the kg of product at the farm gate (live weight, milk, eggs). However, there are some controversies on the best functional unit, either kg of live weight or ha of land, when comparing contrasted production systems, usually the conventional one with more extensive systems (Salou et al., 2017).

Sometimes, dealing with multifunctionality during the goal and scope step is necessary. It occurs when one production process provides different products with different functions. Multifunctionality can be treated through various options: either system expansion or allocation (partitioning) of impacts among co-products. System expansion consists of expanding the system and functional unit to include additional process functions to avoid allocation (FAO, 2018). While ISO standards recommend avoiding allocation in LCA, it is often applied due to the complexity of system expansion (Wilfart et al., 2021). With allocation, inputs and outputs of the system are partitioned over multiple functions according to a partitioning criterion (biophysical relationship, mass, energy content, market price). Allocation is usually applied for feed ingredients that result from the same production process (e.g., soybean oil and soybean meal).

Extensive literature also highlights the issue of allocating the impacts between milk and meat (Wilfart et al. 2021). One usual way to deal with the allocation between milk and meat is to use the energy content of both milk and meat (Aguirre-Villegas et al., 2015). However, it does not account for the inputs needed for milk or meat. Another way is to consider the net energy provided by feed required along the production process to receive milk and meat. As recommended in the ISO standards (ISO, 2006), it refers to the causal relationships between inputs and outputs. Mathematical modeling makes it possible to quantify these causal relationships. For instance, with mathematical modeling, Thoma et al. (2013) calculated biophysical allocation ratios of on-farm burdens between milk and beef co-products. They used the net energy for growth and lactation in order to calculate the allocation ratios of milk and beef. However, they did not include the net energy for maintenance. The study of Aguirre-Villegas et al. (2015) gave an allocation factor of about 99% for milk, while Thoma et al. (2013) found 91.5% with their biophysical approach.

Another example regarding the allocation of impacts among meat co-products is the study of Chen et al. (2017). They proposed a modeling approach based on dynamic systems to assign upstream environmental burdens and raw materials at the farm gate to the livestock co-products at the slaughterhouse. They used a Gompertz function to calculate body protein and then body lipids. From these, they calculated the energy requirements for maintenance and growth, and the partition of energy among tissues was made according to the percentage of protein and lipids in each tissue. Then, they used these energy requirements to calculate biophysical allocation factors among meat products. With this modeling approach, biophysical allocation accounts for the energy cost of building tissues upstream. It makes it possible to propose a rule based on creating the co-products. The studies of Thoma et al. (2013) and Chen et al. (2017) are two examples of mathematical modeling in animal sciences that address the specific issue of allocation in LCA. At the end of the goal and scope, processes considered for the inventory of resources and emissions have been identified.

### Second step—inventory analysis

At this step, the LCA practitioner needs to quantify the resources and emissions associated with each process within the system boundaries and build each process’s life cycle inventories (LCI). For this purpose, it is necessary to define which foreground processes will be quantified using primary data and which upstream or downstream processes will be used, with secondary data available in existing databases. In the literature, foreground data could be taken from surveys, experiments, or mathematical models. Mathematical modeling is powerful in providing primary data using quantitative knowledge of the biotechnical processes. At this step, the benefits of mathematical modeling are the usual ones, i.e., testing various combinations of factors, whatever the context, and access to data unavailable through measurements (e.g., ammonia emissions at the level of a whole farm unit). The contribution of mathematical modeling to the inventory analysis step is further detailed, with various examples in the next section.

### Third step—life cycle impact assessment

This step translates the flows of substances into environmental impacts with a given impact assessment method. An impact assessment method defines the reference substance and the equivalence (characterization) factors for the other substances contributing to each impact category of the process. At this step, various impacts may also be aggregated through normalization to obtain an overall environmental footprint. Experiments and models are necessary to provide reliable methods for life cycle impact assessment, but this is beyond the scope of this publication since it deals with environmental sciences rather than animal sciences.

### Fourth step—result analysis

In this step, the interpretation of the results provided by the impact assessment step could rely on comparing scenarios, contribution analysis, uncertainty, and sensitivity analysis. At this stage, careful analysis of the results is necessary to conclude the environmental damage generated by the system and identify potential strategies for improvement.

Mathematical modeling associated with LCA allows for testing the ecological impacts of a large range of contrasted scenarios, which cannot be reproduced easily using experimental or survey data. The study from Cadéro et al. (2020) illustrates the potential of mathematical modeling associated with LCA to investigate an extensive range of scenarios. They investigated 96 scenarios from a complete factorial design with an individual-based model of the pig fattening unit, simulating the effects of animal characteristics, feeding strategies, and animal management on the technical, economic, and environmental performances. Their model made it possible to cross various factors like batch management, the potential use of a buffer room, the type of feed rationing, the feed sequence plan, the scale at which the feed sequence is applied, and the herd’s health status. It made it possible to highlight that some feeding strategies may compensate to some extent for the effects of the health status.

Uncertainty analysis (UA) and sensitivity analysis (SA) are essential steps of interpretation, as recommended by most guidebooks of LCA, but without clear guidance on how to perform them (FAO, 2018; Curran, 2023). Many sources of uncertainty may disqualify the conclusions drawn from the average values of LCA. Uncertainty analysis and SA are approaches also recommended for performing internal validation of a process-based model before its use (Saltelli et al., 2019).

Different LCA computer software provide tools to run UA given the LCI with Monte Carlo simulations. It makes it possible to assess the uncertainty of the impacts associated to the uncertainty on feed consumption, but it does not consider how the variation of feed consumption could interact with live weight at the farm gate or N emissions. Also, it calculates the uncertainty on the environmental impacts but does not give access to the propagation of uncertainty on the intermediate variables (e.g., N excretion), which can be used to assess the main processes contributing to the uncertainty. Groen et al. (2014) have investigated various methods for UA in LCA (Monte-Carlo sampling, Latin Hypercube sampling). They have raised the following issue. Usually, in UA and LCA, all uncertain parameters included in the analysis are considered independent. If this is not the case, this can lead to either over- or underestimation of the uncertainty on the environmental impacts. Most studies in LCA neglect correlations. These authors mentioned that one way to overcome this issue is to incorporate these dependencies among input parameters by creating a covariance matrix. Here, we argue that another way to deal with this issue is to develop and use a process-based model of the animal production process to perform the UA. With such a model, the internal processes and their interactions are simulated. Consequently, covariances among variables are incorporated, and input parameters are independent. To our best knowledge, there is no example, relying on a process-based model, of uncertainty analysis of environmental impacts for a given animal production process.

The SA is a method close to UA because it is also based on a set of simulations that explore the range of variation of the outputs according to the variation of the inputs. Uncertainty analysis is seen as a preliminary step to explore model behavior, and complementary to SA, it quantifies the contribution of each parameter to the uncertainty of the outputs (Saltelli et al., 2019). SA has an added value in identifying the parameters that need to be quantified to reduce the output uncertainty. Sensitivity analysis can also be used to identify a lever of action to reduce the system’s environmental impact. The studies from Cadero et al. (2018b) and from Kokemohr et al. (2022) illustrate how mathematical modeling associated with LCA makes it possible to investigate the main factors affecting the environmental impacts of livestock production. Cadero et al. (2018b) performed a global SA for conventional pig fattening combining a screening SA method and a variance-based SA method, which was adapted to models with a long simulation time and multiple outputs. They ranked the effects of pig characteristics, farm infrastructure, and farm management and their interactions on technical, economic, and environmental impacts. Kokemohr et al. (2022) also applied global SA of their farm-level optimization model for various European beef production systems. This allowed them to quantify the sensitivity of the outputs to prices (beef, calves, and weaned calves), yields of major roughage crops, and weights of weaned calves and at butchering.

## Specific Contribution of Mathematical Modeling to the Inventory Analysis

The inventory of resources and emissions for LCA in animal production includes the amounts of the feeds consumed at each physiological stage, the consumption of electricity, heat, and tap water, the use of buildings, the emissions of pollutants to the air from the animals and manure in buildings, at pasture and manure storage. It must also specify the production obtained (live weight, milk, eggs) for these resources and emissions. Most of these variables can be estimated using mathematical models developed in animal production. Different mathematical models have been used in the literature while considering the main factors like feeding strategies and performance potential. Most focus on intake, production, and excretion of nutrients with either nutritional models at the animal level, herd or whole-farm models with average animals per category, or even individual-based or agent-based herd or whole-farm models. Some models also specifically focus on the emission of pollutants from animals and per or manure.

### Animal models

Dynamic mechanistic models at the animal level can be helpful in calculating the inventory of emissions for LCA. One approach is to use a nutritional metabolic model of the average animal to simulate feed intake, production (e.g., growth, milk), and the excretion of nutrients. One example of this approach is the use of the InraPorc model (van Milgen et al., 2008) by van Zanten et al. (2015) to perform an LCA of pig production with either a soybean meal-based diet or with a rapeseed meal-based diet for growing-finishing pigs. InraPorc is a dynamic process-based growth model, with body protein and body lipids as main compartments, which simulates feed intake, growth, body composition (protein, lipids), and excretion of N, P, K, Cu, and Zn in response to feed composition (energy, protein, amino acid contents). However, in their study, van Zanten et al. (2015) used InraPorc to calculate only feed intake and growth but not the excretion of nutrients. The N emissions from manure management were calculated using IPCC default values. In the context of their study, where the diets were almost iso-protein, using either default values of N emissions or values calculated from N excretion obtained from InraPorc would have poorly modified climate change impact per kg of live weight. With contrasted feeding strategies, nutrient excretion simulated by InraPorc would differ between scenarios.

Strathe et al. (2015) also built a dynamic growth model of nutrient partitioning, which predicts manure production as the sum of fecal excretion of protein, lipid, dietary fiber, and ash, plus urinary urea excretion and the daily water balance. For dairy cows, Dijkstra et al. (2018) linked a dynamic process-based model of rumen functioning and a static model for intestinal digestion to simulate organic matter, carbon, and N excretion in both feces and urine as a function of diet composition. These models can be further included in a whole-farm model and can also be used to inventory nutrient excretion and calculate emissions from these nutrients in the frame of an LCA.

### Herd or whole-farm models with average animals per category

A second approach is to use models that simulate the animal performance at the livestock farming system level, according to practices, animal characteristics, and per or housing, and calculate emissions from equations with emission factors on nutrient excretion. The modeling results can be aggregated to consider impact at a higher level (regional, national).

Hawkins et al. (2021) investigated the effects of feeding practices on the GHG emissions of dairy cows. They associated attributional LCA and process-based modeling. For this purpose, they used a dynamic model of lifetime productivity, applied to each breed and animal category (cows, bulls, juvenile males, heifers, calves), in which performance depends on the genetic potential of the breed and feeding. The model was used to estimate feed consumption, milk production, and N excretion by dairy cattle in each type of production system and to aggregate at the regional level. It makes it possible to explore the effect of feeding strategies on the productivity of dairy cattle and the resulting impact on GHG emissions.

Many bioeconomic models are available in the literature at the herd or farm level, which can also be used to develop LCI. Indeed, bioeconomic models make it possible to integrate the interactions between mitigation levers and economic context. Kearney et al. (2023) performed a cradle-to-farm gate LCA with a mechanistic whole-farm model simulating pasture-based dairy calf-to-beef production systems, which calculates economic performance and GHG emissions. The herd is divided into several groups based on age, gender, and production purpose. With this model, they evaluated the economic and environmental impacts of individual or combined GHG mitigation practices. The Integrated Farm System Model (IFSM) (Rotz et al., 2025) is a bioeconomic model of beef and dairy sytems, which provides the LCI obtained from process-based modeling. It shares some features with the model from Kearney et al. (2023): average animal representation per stage, mechanistic modeling of animal response to feeding. Similarly, Kokemohr et al. (2022) performed an LCA of some European beef production systems, using the FarmDyn model to generate the LCI for the inputs and outputs. FarmDyn is a bioeconomic whole-farm model that maximizes profit while considering variability in prices, yields, and animal performance. These authors argued that in the LCA context, optimization models can provide insights into changes in agricultural systems’ environmental performance due to farmers’ adaptation to changing conditions such as price or yield changes (Veysset et al., 2010).

Using this kind of bioeconomic model is relevant when the effects of the economic context on the effectiveness of mitigation strategies are foreseen. There are also situations where other types of interactions can occur with mitigation strategies. In particular, various feeding and animal management strategies have been investigated as mitigation strategies, but most are applied at the group level and interact with the variability of requirements and potential among animals.

### Individual- or agent-based models at the herd or whole-farm levels

Some studies showed interest in considering the variability of animal performance. Brossard et al. (2009) estimated the five main parameters of the InraPorc model for 193 pigs and further simulated the performances of these pigs with InraPorc v 1.0.4.0 with various feeding programs. They showed that the percentage of pigs whose digestible amino acid requirements are covered at the end of each feeding phase decreases when the number of feeding phases increases, resulting in degraded performance at the group level. Villalba et al. (2006) developed a model simulating dairy cows’ productive and reproductive performance. They explored the difference between simulating complex groups of animals with different initial characteristics and calving dates versus using average animal characteristics. Considering variability among a population allowed them to simulate the behavior of real batches, where animals are grouped with homogeneous feeding and reproductive management.

Therefore, few individual- or agent-based models investigating the interactions between practices and the variability among the animals have been developed and applied in an LCA context. Monteiro et al. (2016) fitted parameters of the InraPorc model with InraPorc v1.6.5.8. for a batch of growing pigs in both a French and a Brazilian context and then simulated each pig’s intake, growth, and nutrient excretion under various feeding strategies. These authors used these outputs to develop the LCI for a cradle-to-farm gate LCA, which tests the effects of production context and feeding strategies. In particular, they highlighted the reduction of N excretion in daily individual multiphase feeding vs. daily group multiphase feeding. Cadéro et al. (2018a) developed an individual-based model of a pig-fattening unit simulating the interaction between animal characteristics, management practices, and housing. The model provides the LCI for a cradle-to-farm gate LCA. It calculates technical, economic, and environmental performance at individual, group or batch, and whole-unit levels. Animals are represented using the InraPorc model. Practices are implemented at the farm, batch, and animal levels. Implementing management practices, including batch management and delivery to slaughterhouse, the variability among animals, and housing, makes it possible to evaluate the consequence of farmer practices in interaction with real farm constraints on the environmental impacts of the production.

**Figure 2.**
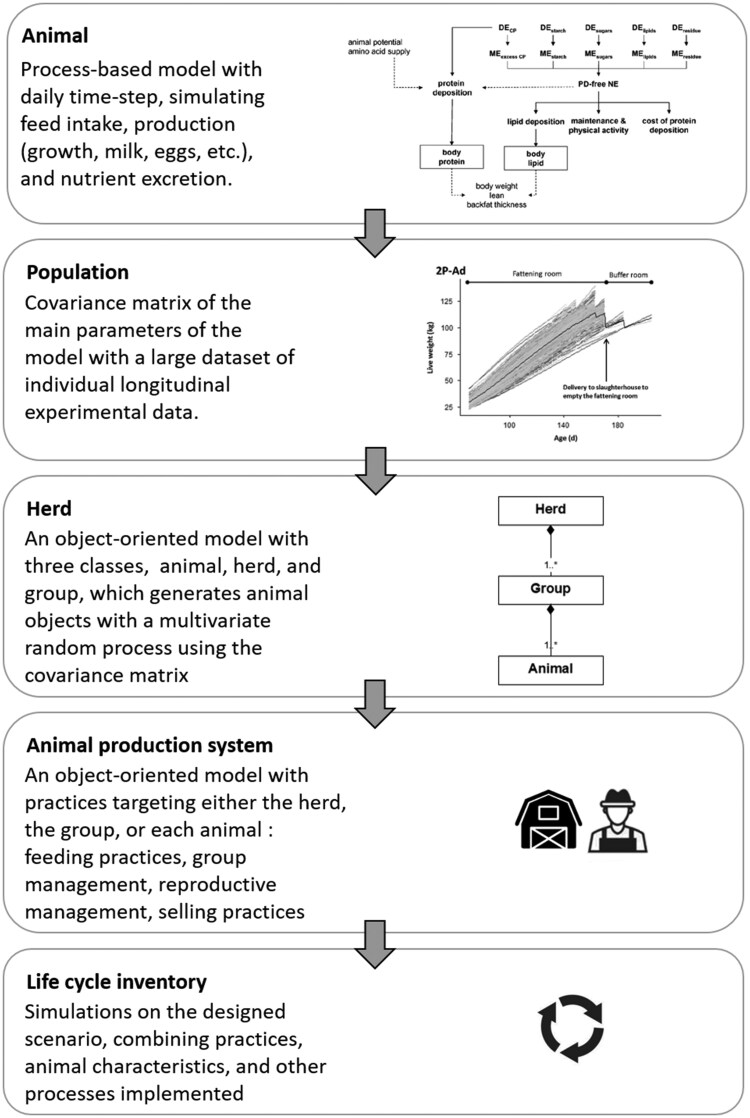
Workflow in developing an individual-based model of an animal production system for further use in producing life cycle inventory in a cradle-to-farm gate LCA. The illustrated animal model comes from van Milgen et al. (2008), while the displayed individual growth data comes from Cadéro et al. (2018a).

This example of associating an individual-based model of a livestock production system with the LCA framework is innovative and almost unique in the literature. In this approach, the authors obtained a stochastic, individual-based, discrete-event model that captures the complexity of the system to produce the LCI. The overall development process (Figure 2) could be applied to other livestock production systems. It could be described with five steps that highlight a bottom-up approach, from the animal to the life cycle:

- The workflow starts with a process-based model at the animal level, representing the animal production potential and the possible limiting effect of feeding on achieving the potential performance. At this stage, it is also essential to identify the most sensitive parameters of the model, which could be fitted individually. For further use in LCA, this model should also calculate the excretion of nutrients (N, P).
- The second step relies on experimental data. The aim is to be able to estimate the individual values of the most sensitive parameters to generate a virtual population. For this purpose, a large dataset with individual longitudinal experimental data of feed intake and production in non-limiting conditions is used to fit the main parameters of each animal’s process-based model. These individual values of parameters are then processed to obtain their covariance matrix. Vautier et al. (2013) describe this step in detail for growing pigs.
- The third step aims to generate the individuals composing the whole herd and allocate them in groups. To handle individual interactions, we recommend using object-oriented programming. Object-oriented programming is defined as a programming paradigm based on the concept of objects described in classes. Objects can be built from classes, which are the equivalent of blueprints, and contain specific data and actions they can perform. These objects are used in programs to represent interactions between system components. In the approach, it is useful to define three classes: one class for the animal level, one for the whole herd level, and one for an intermediate level corresponding to a management entity. They will make it easy to implement practices that may apply to different levels. The animal objects are generated using a multivariate random process and the covariance matrix.
- The goal of the fourth step is to add practices and their execution at herd, group, and individual levels to upscale the livestock farming system level. During the simulation, a calendar of events is built to organize the dynamic of events linked to practices. The practices can be implemented following an iterative process, with validation by expert knowledge at each iteration step. The consistency and effective functioning of the model is checked during the development process. Various practices can be implemented: feeding practices, group management, reproductive management, and selling practices.
- The last step is the calculation of the values that will fill the LCI. Simulations are run on the designed scenario, combining practices, animal characteristics, and other processes implemented. The amount of product (live weight at farm gate, milk at farm gate) obtained and emissions from animals and manure for housing and storage (CH_4_, N_2_O, NH_3_, and NO_x_) are calculated. Inputs and outputs of the LCI at the farm level are calculated by aggregating values from the animal level.

### Models of emissions from animals and manure

The most well-known guidelines to conduct LCA in animal production and per or to calculate the emissions of pollutants for national inventories (FAO, 2016a, 2016b, 2016c, 2018; IPCC, 2019; EMEP/EEA, 2016) identify three levels of complexity when calculating the emissions of pollutants from animals and from manure.

The Tier 1 method is a simplified empirical approach that uses default emissions to distinct animal categories. Consequently, it is impossible to investigate mitigation strategies except for reducing animal numbers (Kebreab et al., 2016). The Tier 2 method is the most widely used in the context of LCA in animal production. It captures some of the effects related to diet composition, but in general, it is limited to the effects of feed intake, energy, and N contents. The recommended methodology, when available, is the Tier 3 approach, which uses process-based models to capture as many interacting factors as possible. Therefore, many of these models have been developed. Tedeschi et al. (2022) reviewed existing methods to measure and per or estimate enteric methane, including these IPCC tier-based approaches, which progress from using default emission factors (Tier 1) to more sophisticated feed and animal characterization (Tier 2) and finally to region-specific models (Tier 3). They highlighted the usefulness of mechanistic models in estimating the methane conversion factor (as a proportion of gross energy intake) for inventory purposes compared to empirical models. Vibart et al. (2021) also highlighted the need for process-based models simulating rumen function to capture the effect of dietary mitigation strategies. However, as Tedeschi et al. (2022) emphasize, modeling of CH_4_ emissions from manure is particularly challenging due to complex data requirements and parameterization issues. Current mechanistic models often fail to account for microbial responses to variations in manure temperature, substrate availability, age, management systems, and the distinction between short- and long-term responses to environmental changes (Tedeschi et al., 2022). These models exhibit high uncertainty with reported CH_4_ emissions from liquid manure in anaerobic lagoons and slurry storage systems ranging from 368 ± 193 to 101 ± 47 kg CH_4_ per head/year, respectively (Tedeschi et al., 2022). While some models function as components of whole-farm approaches, and others simulate specific manure systems, Tedeschi et al. (2022) note that there remains “a need for simpler models that use fewer input parameters than mechanistic models but can adequately represent C and N flows dynamically and are sensitive to most of the factors influencing GHG emissions.” Unfortunately, few mathematical models have been successfully developed and applied following these balanced principles, creating a significant gap in our ability to quantify manure emissions in LCA contexts accurately.

Process-based models have been developed to estimate the impact of various factors, including mitigation strategies, on ammonia emissions. One example is the study by Mendes et al. (2017) that proposes a mechanistic model of ammonia emissions at the barn level based on kinetics and using ordinary differential equations. This model dynamically simulates the effects of floor type, floor scraping and flushing, and manure acidification on NH_3_ emission reduction factors from dairy cattle barns.

One hybrid model has recently been proposed to simulate ammonia emissions from dairy manure during storage (Genedy et al., 2024). It associates process-based modeling with neural networks. Their study identified that it is challenging to incorporate into the model events (surface crusting, snow cover) that influence emissions but have no defining equations. Therefore, they integrated machine learning into their model to create solutions for systems with no existing formulas and equations or written rules. Their approach improved the process-based models’ generalization and accuracy in estimating ammonia losses from stored liquid dairy manure. However, many of these emissions models are not used in the context of LCA.

In the frame of a cradle-to-farm gate LCA, whole-farm models are generally adopted to quantify emissions. However, these farm-scale models rely heavily on Tier 2 emission factors and empirical relationships to estimate emissions. Nonetheless, this approach does not always capture the detailed underlying processes and variations in the drivers that impact emissions. Few attempts have been made to integrate processes at the farm level to capture the effects of various factors, in particular diet, on whole farm emissions, including enteric methane and manure emissions. Among them, Ouatahar et al. (2024) proposed a cascade of process-based models to simulate the effects of diets on enteric and manure emissions, with the first model predicting enteric methane emissions and C and N excretions, the second one predicting emissions from manure at housing, manure storage, and field application, and the third one dealing with soil emissions. For enteric methane and urine and feces C and N excretions, they developed a system dynamics model (Tedeschi, 2023) with ordinary differential equations that describe the change in time of pools of the substrate, micro-organisms, and microbial end-product present in the rumen and large intestine. For the emissions from manure at housing, manure storage, and field application, it applies a process-based model designed to simulate the biogeochemical cycles of C, N, and P, based on the principles of thermodynamics and reaction kinetics, controlled by various environmental factors such as temperature, pH, and moisture. Their approach highlighted a considerable variation in emissions per hectare or head basis and across different farm components and categories of animals, showing that more simple approaches like Tier 1 and Tier 2 cannot capture the variations.

## Mathematical Modeling and LCA to Support Mitigation of Impacts: How to Move Forward?

The preceding discussion has demonstrated how process-based modeling can contribute to various components of LCA, particularly in developing comprehensive LCI. However, to be fully operational, LCA, assisted by mathematical modeling in animal production, must conclude to support decisions and policies. Process-based modeling must support evidence-based decision-making and policy development to achieve maximum utility in animal production systems. This section examines recent methodological advances that integrate LCA with complementary modeling approaches, focusing on two key applications: 1) the development of mitigation strategies with LCA associated with optimization models or 2) the formulation of strategies for genetic selection. Additionally, this section addresses the inherent limitations of LCA methodology and explores how integrating with modeling approaches can enhance investigations of mitigation strategies at national or supranational scales.

### Optimization models to support management decisions

Mathematical modeling associated with LCA has the potential to test various strategies for the mitigation of the environmental impacts of animal production, as illustrated in previous sections. However, there are some attempts in the literature to go beyond this approach by developing and solving optimization problems to support the design of strategies to reduce environmental impacts while maintaining economic performance.

The first approach is to address this issue at the feed level. For instance, Méda et al. (2021) evaluated the environmental impacts of pig production considering two feed formulation methods: a baseline least-cost formulation (LCF) and a multiobjective formulation (MOF) incorporating the environmental impacts of feed production. For pigs, they coupled their optimization model of feed formulation to the individual-based pig fattening model by Cadéro et al. (2018a) and tested three scenarios of feeding sequences. The use of MOF decreased the environmental impact of pigs at the farm gate and decreased the gross margin compared to the use of LCF. They concluded that multiobjective optimization should consider feed cost and environmental and economic performances at the farm gate.

Overall, various examples of bioeconomic models maximize the profit in an animal production system and then calculate the resulting environmental impacts through LCA (Mosnier et al., 2017; Davoudkhani et al., 2020). However, these models do not help identify mitigation strategies. Bioeconomic models optimizing the environmental impacts obtained through LCA and the profitability of animal production systems are very scarce in the literature. This situation is probably due to the methodological complexity of addressing multiobjective optimizations at group, herd, or farm levels. One of the rare examples is the study by Marques et al. (2022), who proposed a bioeconomic model to assess the economic and environmental tradeoffs in diet formulation for feedlots in France. They addressed the multiobjective problem by adding to their profit-maximizing function an epsilon-constraint on a weighted sum of normalized environmental impacts, varying between an upper bound determined by solving the problem without the constraint and a lower bound by minimizing the weighted sum of implications. With this approach, they found in their context that the cheapest mitigation lever is to reduce the LCA footprint of the feed and feeding and that further mitigation should involve modification of the diet composition to minimize methane emissions.

### Individual-based models to support genetic selection

Genetic selection is the purpose of many studies, as a lever to mitigate the environmental impacts of animal production. For ruminants, the focus is primarily on the reduction of enteric methane production, and recent promising results have shown the effectiveness of genetic selection in dairy production to reduce the emissions of enteric methane (González-Recio et al., 2020).

Few articles on dairy cattle have also explored the effect of production and fitness traits on GHG emissions, but without including the emissions from feed production. For instance, Pryce and Bell (2017) investigated the consequences of previous and current genetic selection practices on responses of carbon emissions to selection for key traits included in the Australian national breeding objective. For that purpose, they used the model from Bell et al. (2013) to predict the carbon dioxide equivalent emissions per unit change of these traits while holding all other characteristics constant. This model only considers enteric and manure CH_4_ and direct and indirect N_2_O from the storage and application to the land of manure per kilogram of milk solids.

Various attempts have been made in pig production to elucidate the effectiveness of genetic selection on production traits to reduce the environmental impacts of the production. Contrary to most studies in ruminant production, the scientific community has predominantly adopted the LCA approach, considering not only GHG emissions but including all the processes contributing to the impacts from the resources used for crop production up to manure storage (Ottosen et al., 2020; Soleimani and Gilbert, 2020). This is adequate in pig production, where feed production significantly contributes to environmental impacts, including GHG emissions.

**Figure 3.**
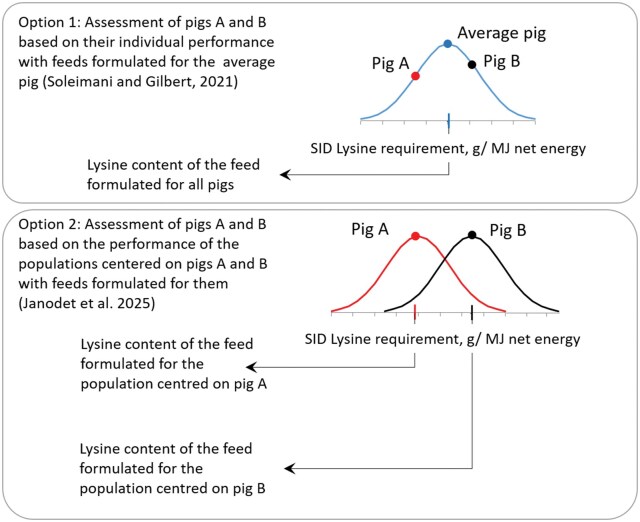
Comparison of the methodological approaches developed by Soleimani and Gilbert (2021) (Option 1) and by Janodet al. (2025) (Option 2) to perform individual life cycle assessment in pig production (adapted from Janodet et al. (2025).

A challenge in these approaches is to simulate the interaction between the genetic traits at the individual level and the farm environment to represent the impact of genetic selection at the whole-farm level. In other words, the goal is to phenotype each individual by evaluating the environmental impacts, usually calculated at the production system level. This objective could be achieved with individual-based modeling. Soleimani and Gilbert (2020, 2021) have proposed a first attempt toward this direction. They developed a trait-based individual LCA model that has the following characteristics: 1) individual pig profiles are fitted with InraPorc v1.7.1.0 using experimental in vivo data, 2) individual performance of growth, intake, and excretion is simulated with the InraPorc software, 3) individual performance is used to calculate the emissions from animals and manure, and 4) these simulated data are incorporated into individual LCI to calculate individual environmental impacts further. They concluded that animal selection for feed efficiency and environmental optimization of the diet have significant potential to reduce environmental impacts on pig production. However, in Soleimani and Gilbert (2020, 2021), the feeding strategies were based on the population requirements, leading to bias in the expression of performance by the pigs (Figure 3). More recently, Janodet et al. (2025) (Figure 3) proposed another method of individual environmental assessment to address this problem. They developed a methodology to phenotype each pig via the evaluation of the environmental impacts obtained for a pig production system if this pig becomes the average animal of the population. For each “parent” pig of the initial population, they generated one “son” population centered on its individual characteristics. For each “son” population, they formulated feeds with digestible amino acid contents adapted to the requirements of the “parent” pig. The performances and environmental impacts of each “son” population were simulated with an individual-based model adapted from Cadéro et al. (2018a). The individual environmental impacts of each “parent” pig were calculated as the environmental impacts of the “son” population simulated (per kg live weight).

To our knowledge, this type of approach has not been developed so far for ruminants. Few examples of individual-based models have been developed to address the combined effects of genetic selection and feeding strategies. However, they are not associated to date with life cycle assessment to explore the effects of these combined levers on the environmental impacts of animal production. For instance, Bouquet et al. (2022) coupled genetic and mechanistic models of a grass-based dairy cow system to test the sensitivity of genetic traits for milk production and feed efficiency. The mechanistic model simulates individual phenotypic trajectories over a cow’s lifetime for milk production, dry matter intake, body mass components, including body reserves, as well as the timing of estruses and success of reproduction events (Puillet et al., 2016). Their scenarios crossed feeding strategies differing in intake restriction and heritability of the genetic traits. They concluded that this approach is interested in exploring new traits for genetic improvement and defining new breeding strategies under various environmental scenarios. Environmental impacts calculated through LCA could be some of these new traits explored with this model.

### Upscaling to identify trajectories for animal production systems—what is LCA’s role?

Drawing conclusions from LCA to support policies and decisions at the national level remains challenging. It begs the questions: are LCA methodologies sufficiently robust to capture all the nuances related to livestock production, nutrient cycling, and greenhouse gas emissions with the reliability required for policy development? Are the findings obtained with LCA sufficiently robust to apply a given mitigation measure globally? Usually, studies that try to identify sustainable pathways for animal production systems do not rely (only) on the LCA approach. del Prado et al. (2025) have recommended the use of different methods, including LCA, at animal, farm, national, and supranational scales for quantifying the GHG emissions abatement allowed by the use of anti-methanogenic feeds additives in the livestock sector. Indeed, they identified that using anti-methanogenic feed additives can lead to reduced feed digestibility, which may increase methane emissions from manure and the overall performance of ruminants. Feedback loops that may offset the benefits identified with an LCA should be identified and addressed as much as possible.

Many studies also argue that big changes should be considered to address the huge environmental challenge of livestock production. These studies raise questions about the development of alternative production systems (van Hal et al., 2019) and the extent to which specialization and per or intensification of livestock production systems should be reconsidered (Billen et al., 2018; Faverdin et al., 2022). Most of these studies do not rely on LCA. Faverdin et al. (2022) explored the issue of either specializing and or intensifying milk and beef production. For this purpose, they compared results obtained at herds, farm, and country levels with both LCA and mathematical modeling at the national level. They first reported that LCA studies showed that increasing milk production per cow in specialized dairy systems improves feed efficiency and reduces the carbon footprint of milk at farm level. With their demographic model of the cattle sector at national level, while respecting the livestock demographic constraints and maintaining constant milk and meat production levels, they also simulated the consequences of an improved milk production at animal level. They found that if the cattle milk-to-meat production ratio remains unchanged in order to answer to the demand, an increase in dairy cow productivity must be compensated for by a simultaneous increase in the number of beef cows, since the improvement of dairy cow productivity results in a lower meat production from the dairy sector. With this approach, they highlighted that this mechanism can offset the expected mitigation of GHG emissions allowed by the reduction of dairy herd. Although previous LCA have not been able to capture these effects, their modeling approach at national level, investigating GHG emissions, is not so far from an LCA since they account also for the upstream emissions in a holistic approach, and they use the characterization factors of life cycle impact assessment to aggregate the different types of GHG emissions. In this context, the LCA framework could have been used but with system boundaries considering the whole livestock sector at national level while considering an appropriate functional unit (e.g. one inhabitant fed in beef and milk products during 1 yr).

Furthermore, integrating precision livestock farming (PLF) technologies with mathematical modeling represents another promising frontier for enhancing the environmental assessment of animal production systems. The continuous nature of PLF-derived data enables dynamic modeling approaches that capture temporal variations in animal performance and environmental responses, which are typically overlooked in static LCA approaches. Indeed, PLF technologies—including wearable sensors, automated feeding systems, and real-time monitoring devices—generate high-resolution, individual-level data on feed intake, growth, health status, and behavior patterns. These data streams could significantly reduce uncertainty in modeling by providing accurate inputs for parameterization and validation. In addition, PLF could facilitate the implementation of mitigation strategies identified through LCA by enabling precision management of individual animals based on their environmental performance profiles, and also serving as validation of the LCA predictions given the amount of data that can be collected. This synergy between PLF and model-enhanced LCA creates the foundation for adaptive management systems that continuously optimize environmental and economic performance across scales. Future research should explore methodological frameworks, from individual animals to national livestock sectors, that effectively incorporate PLF-derived data into LCA to support evidence-based policies for sustainable livestock production.

## Conclusion

Mathematical modeling in animal production brings LCA far beyond what could be accomplished with surveys and experiments. It gives LCA the ability to precisely estimate the effects of interacting factors of variation on the consumption of resources and the emissions occurring along the life cycle. It also makes it possible to investigate numerous scenarios in different production contexts. Its main contribution is to the life cycle inventory stage, but it can provide a valuable approach to deal with multifunctionality and draw conclusions from scenario analysis and uncertainty and sensitivity analyses.

Most process-based models available so far, and used in LCA, target either the prediction of animal performance and excretion of nutrients or the prediction of emissions given a level of performance and excretion. There is probably an interest in the association of both models to ensure all feedback loops occurring between animal nutrition, performance, and emissions from animals and manure are fully considered before assessing mitigation options. When dealing with usual levers considered by animal scientists, like feeding strategies and genetics, individual-based models appear as promising tools because they make it possible 1) to address both the individual animal scale and the farm scale levels and 2) to account for farming practices that target either the individuals or the group.

In the future, researchers should consider further developments targeting, for instance, the identification of mitigation strategies through optimization models, genetic selection for reduced environmental impacts, or national and supranational scales should be considered. The emerging field of precision livestock farming will offer significant potential for more responsive and adaptive environmental management frameworks while providing real-world validation of LCA predictions. For these purposes, they will need further methodological frameworks associating mathematical modeling in animal production and LCA.

## Abbreviations:

CH4: methane
EEA: European Environmental Agency
EMEP: European Monitoring and Evaluation Programme
FAO: Food and Agriculture Organization
GHG: greenhouse gas
IPCC: Intergovernmental Panel on Climate Change
ISO: International Organization for Standardization
LCA: life cycle assessment
LCF: least-cost formulation
LCI: life cycle inventory
MOF: multiobjective formulation
N_2_O: nitroux oxide
NH_3_: ammonia
NOx: nitrogen oxides
PLF: precision livestock farming
SA: sensitivity analysis
UA: uncertainty analysis
